# Preparation and Performance Evaluation of a Biodegradable Gel for Deep Coal Reservoir Drilling Fluids

**DOI:** 10.3390/polym18141748

**Published:** 2026-07-17

**Authors:** Jiang Xin, Xinyu Hao, Zongkai Qi, Gang Chen, Wei Wang, Zenglong Wang, Jinliang Han, Jiafeng Jin, Gengshu Wang

**Affiliations:** 1 China United Coalbed Methane National Engineering Research Center Company Limited, Beijing 100095, China; 2 PetroChina Coalbed Methane Company Limited, Beijing 100028, China; 3 School of Petroleum Engineering, China University of Petroleum (East China), Qingdao 266580, China

**Keywords:** deep coalbed methane, biodegradable plugging agent, rock mechanics, wellbore stability

## Abstract

Deep coal reservoirs are characterized by abundant microfractures and cleats, resulting in frequent wellbore instability during long horizontal drilling operations. To address this issue, a biodegradable gel plugging agent (XZ) was synthesized from acrylamide (AM) and carboxymethyl cellulose (CMC) via an orthogonal crosslinking strategy. The gelation behavior, swelling capacity, rheological properties, mechanical strength, thermal and salt resistance, plugging performance, self-degradation characteristics, reservoir protection capability, and drilling-fluid compatibility of XZ were systematically evaluated. The results showed that XZ formed a stable three-dimensional crosslinked network with good thermal stability (T_0_ = 116 °C, T_1_/_2_ = 445 °C). The synergistic effects of covalent and ionic crosslinking endowed the gel with high swelling capacity, mechanical strength, and plugging ability. XZ exhibited excellent temperature and salinity tolerance, with a degradation rate exceeding 90% after 5 days. After degradation, the filter cake became significantly thinner and core permeability recovery reached approximately 80%, demonstrating effective reservoir protection. Moreover, XZ significantly increased drilling-fluid viscosity, reduced fluid loss, and enhanced plugging performance. The XZ-based drilling fluid system integrates efficient plugging, self-deplugging, and reservoir protection functions, providing a promising solution for safe and efficient drilling in deep coalbed methane reservoirs.

## 1. Introduction

China possesses abundant coalbed methane (CBM) resources, particularly deep structural coal-measure gas resources with enormous development potential. According to national CBM resource assessments and studies in the field of natural resources, the geological reserves of CBM in China exceed 30 × 10^12^ m^3^, ranking among the highest in the world [1,2]. These resources are primarily distributed in the Ordos, Qinshui, Sichuan, Junggar, and Turpan–Hami basins. Deep structural coal-measure gas reservoirs are generally characterized by great burial depths, high formation pressures, and extensively developed natural fractures and cleat systems, making them a crucial strategic replacement resource for increasing unconventional natural gas reserves and production in China. With the rapid advancement of deep CBM and coal-measure gas development, drilling-related challenges such as wellbore instability, pipe sticking, and excessive borehole enlargement have become increasingly severe, significantly hindering safe and efficient drilling operations. Therefore, the development of enhanced wellbore-stabilizing and anti-collapse drilling fluids for coal-bearing formations is of considerable theoretical significance and practical engineering value.

Degradable gels are a class of polymeric materials capable of undergoing network disintegration or chemical degradation under specific environmental conditions [3,4]. Owing to their unique combination of effective sealing performance and subsequent self-removal capability, degradable gels have demonstrated broad application prospects in lost-circulation control, reservoir protection, temporary plugging during hydraulic fracturing, and profile control/water shutoff treatments. As unconventional oil and gas exploration continues to expand into deeper and more challenging formations, issues such as wellbore instability, reservoir damage, and drilling fluid losses have become increasingly prominent, placing higher demands on plugging materials with the characteristics of strong sealing capacity, minimal formation damage, and controllable removal. Consequently, degradable gels, which can effectively seal fractures during drilling and subsequently degrade after completion, have attracted growing research interest.

Research on degradable gels was initiated relatively early in foreign countries and has mainly focused on reservoir protection and temporary plugging fracturing technologies [5,6,7]. By introducing degradable structural units such as ester bonds and acetal bonds, researchers have developed intelligent gel systems responsive to temperature, pH, and enzymatic environments, enabling controlled degradation under specific reservoir conditions. Meanwhile, advanced strategies including double-network crosslinking, multiple crosslinking mechanisms, and nanomaterial reinforcement have been employed to enhance the mechanical strength, thermal stability, salt tolerance, and pressure-bearing capacity of gels, thereby meeting the requirements of complex reservoir environments. In recent years, the synergistic design of high-strength plugging performance and controllable degradation behavior has emerged as a major research direction worldwide.

Domestic studies have primarily focused on drilling fluid loss prevention, reservoir protection, and the development demands of unconventional oil and gas resources [8]. Various gel systems have been constructed using polyacrylamide, cellulose derivatives, and natural polymers through chemical crosslinking, ionic crosslinking, and physical entanglement techniques. Significant progress has been achieved in improving water absorption and swelling properties, plugging effectiveness, and adaptability to high-temperature and high-salinity environments. Some studies have further realized controlled-release degradation and self-removal functions, thereby mitigating reservoir damage. However, most existing investigations have been conducted for conventional reservoirs or shallow-to-medium depth formations, while research specifically targeting the harsh conditions of deep coal-measure gas reservoirs remains limited.

Deep coal-measure gas reservoirs are typically characterized by great burial depths, elevated temperatures, high formation pressures, extensively developed fracture systems, and strong stress sensitivity. During drilling operations, plugging materials must rapidly penetrate microfractures and pore spaces to establish a high-strength sealing barrier that prevents drilling fluid invasion and maintains wellbore stability. Simultaneously, these materials are expected to degrade efficiently after drilling and completion operations, enabling blockage removal and flowback recovery while preserving reservoir permeability to the greatest extent possible. Therefore, current degradable gel systems still face several challenges, including the difficulty of simultaneously achieving strong plugging performance and efficient degradation, insufficient thermal and salinity resistance, poor controllability of degradation behavior, and an incomplete understanding of reservoir protection mechanisms [9,10].

To address these challenges, a novel degradable gel was developed in this study with excellent plugging performance, superior thermal and salinity tolerance, autonomous degradability, and effective reservoir protection capability. The gelation, plugging, and degradation mechanisms were systematically investigated. The proposed material provides a promising solution to the long-standing contradiction between strong plugging performance and low formation damage during deep coal-measure gas drilling operations, thereby offering technical support for safe and efficient drilling in deep coal-measure gas reservoirs.

## 2. Experimental Methods

### 2.1. Materials

The monomers used in this study included acrylamide (AM, purity ≥ 99%, analytical grade, purchased from Aladdin Reagent Co., Ltd., Shanghai, China), sodium carboxymethyl cellulose (CMC, degree of substitution 0.7–0.9, industrial grade, purchased from Sinopharm Chemical Reagent Co., Ltd., Zibo, China), and polyvinyl alcohol (PVA, degree of alcoholysis 87–88%, analytical grade, purchased from Macklin Biochemical Co., Ltd., Shanghai, China). The crosslinking agent N,N′-methylenebisacrylamide (MBA, purity ≥ 99%, analytical grade) was obtained from Aladdin Reagent Co., Ltd. (Shanghai, China), while ammonium persulfate (APS, purity ≥ 98%, analytical grade) was used as the initiator and purchased from Sinopharm Chemical Reagent Co., Ltd. (China). Deionized water prepared in the laboratory, with a conductivity lower than 1 μS/cm, was used throughout all experiments.

### 2.2. Synthesis of the Degradable Plugging Agent

The degradable gel XZ was synthesized using a stepwise crosslinking method. First, PVA was dissolved in deionized water to form a homogeneous solution. Subsequently, AM and the crosslinking agent were thoroughly mixed, and a stable covalently crosslinked AM network was constructed through free-radical polymerization initiated by APS [11,12,13]. After completion of the polymerization reaction, CMC and Ca^2+^ were introduced to form a secondary network via ionic crosslinking and intermolecular physical entanglement.

**Figure 1:**
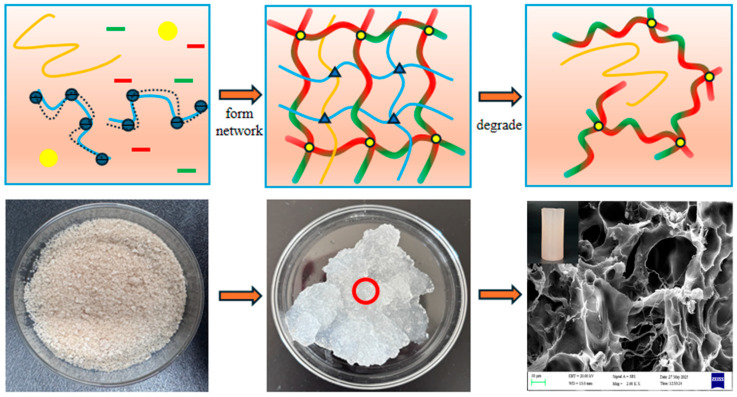
Schematic illustration of the synthesized degradable gel XZ.

The resulting material was cured to obtain the degradable gel XZ with a multiple-network structure. In this system, the covalently crosslinked AM network generated through free-radical polymerization serves as the primary framework, providing mechanical strength and structural integrity. Meanwhile, the ionic crosslinking between CMC and Ca^2+^ forms a secondary network, while the physically entangled PVA molecular chains further reinforce network stability. The synergistic effect of these multiple-network structures endows the gel with excellent water-swelling capacity, mechanical strength, and plugging performance. The preparation procedure of the degradable gel XZ is illustrated in Figure 1.

### 2.3. Characterization

(1) Fourier Transform Infrared Spectroscopy (FTIR)

The chemical structure and functional groups of the prepared degradable plugging agent were characterized using a Fourier transform infrared spectrometer (Bruker Vertex 70, Germany). Prior to testing, the samples were thoroughly dried, ground into fine powders, and uniformly mixed with potassium bromide (KBr). The mixtures were then compressed into transparent pellets for analysis. The functional group composition and crosslinking characteristics of the material were identified by analyzing the positions and variations in the characteristic absorption bands [14].

(2) Proton Nuclear Magnetic Resonance (^1^H NMR) Analysis

The molecular structure of the degradable plugging agent was characterized using proton nuclear magnetic resonance (^1^H NMR) spectroscopy [15]. The chemical shifts and peak patterns of characteristic hydrogen atoms were analyzed to determine the polymer chain structure and functional group composition, thereby verifying monomer polymerization and the formation of the crosslinked network.

(3) Rheological Measurements

The rheological properties of the degradable plugging agent were evaluated using a HAAKE rheometer. Key parameters, including viscosity, storage modulus (G′), and loss modulus (G″), were measured to assess the viscoelastic behavior and structural stability of the gel. The mechanical response of the gel network after formation was analyzed to provide a basis for evaluating its plugging performance [16].

(4) Scanning Electron Microscopy (SEM)

The microstructures of the degradable plugging agent and drilling-fluid filter cakes were examined using a scanning electron microscope (TESCAN VEGA GMS). Before observation, the samples were dried and sputter-coated with gold to enhance electrical conductivity [17]. The surface morphology, pore distribution, and filter-cake compactness were analyzed to investigate the network structure of the plugging agent and its effect on improving filter-cake integrity.

(5) Evaluation of Gel Strength and Toughness

The strength of the gel was evaluated based on the relative mechanical strength of the pre-crosslinked gel after water absorption. Specifically, a certain amount of water-saturated pre-crosslinked gel particles was subjected to static observation at a constant temperature, and the deformation behavior of the swollen particles under compression was recorded. The strength and toughness were classified according to the rating criteria listed in Table 1. This rating system provides a semi-quantitative, preliminary assessment that is convenient for rapid screening of gel formulations; it is not intended to replace instrumental characterization. The primary quantitative mechanical characterization is provided by the rheological measurements (storage modulus G′ and loss modulus G″) presented in Section 3.1 (3). Based on this classification system, the swollen gel exhibited a strength rating of Grade D, corresponding to a moderately elastic and deformable gel, and a toughness rating of Grade D, indicating good toughness and resistance to fragmentation.

## 3. Results and Discussion

### 3.1. Characterization

(1) Fourier Transform Infrared Spectroscopy (FTIR)

**Figure 2:**
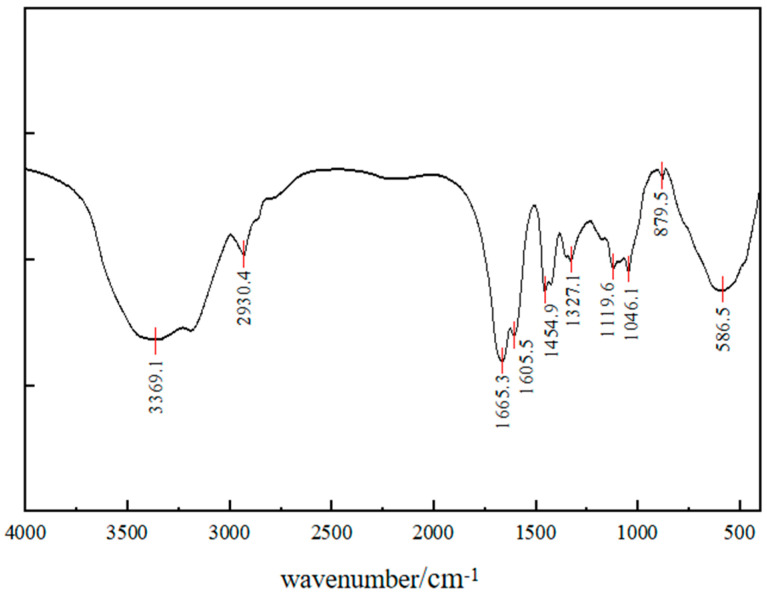
FTIR spectra of the degradable gel XZ.

As shown in Figure 2, the degradable plugging agent exhibited a broad and intense absorption band at 3369 cm^−1^, which is attributed to the stretching vibrations of hydroxyl (–OH) and amino (–NH) groups. The absorption peak at 2934 cm^−1^ corresponds to the stretching vibrations of –CH_2_– and –CH_3_ groups. Characteristic peaks observed at 1653 cm^−1^ and 1605 cm^−1^ are assigned to the C=O stretching vibration and N–H bending vibration of the amide group (–CONH_2_) [18], respectively, indicating the presence of polyacrylamide (PAM) structures within the gel network. The absorption bands at approximately 1327 cm^−1^ and 1151 cm^−1^ are associated with the vibrations of C–N and C–O bonds, respectively, while the peak at 1046 cm^−1^ is attributed to ether (C–O–C) linkages and/or hydroxyl-containing alcohol groups, confirming the incorporation of CMC and PVA into the gel system. In addition, the absorption peaks at 879 cm^−1^ and 586 cm^−1^ are related to the skeletal vibrations of the polymer backbone. Overall, characteristic absorption bands corresponding to hydroxyl, amide, and ether functional groups were simultaneously detected in the FTIR spectrum, demonstrating the successful incorporation of AM, CMC, and PVA into the gel matrix. These results confirm the formation of a stable multiple-crosslinked network structure, which provides the structural basis for the gel’s water-swelling behavior, plugging performance, and controlled degradability.

(2) Proton Nuclear Magnetic Resonance (^1^H NMR)

**Figure 3:**
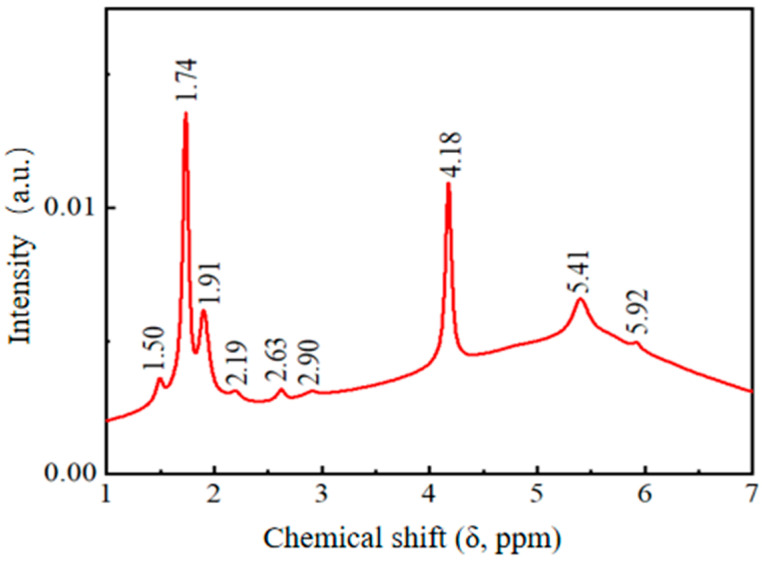
^1^H NMR spectrum of the degradable gel XZ.

As shown in Figure 3, the ^1^H NMR spectrum of the degradable plugging agent exhibits a prominent resonance peak at δ = 1.74 ppm, which is attributed to the proton signals of the –CH_2_– and –CH– groups in the polyacrylamide (PAM) backbone. Several resonance peaks observed in the range of δ = 2.19–2.90 ppm are assigned to the protons on carbon atoms adjacent to the amide groups, confirming the successful polymerization of the acrylamide (AM) monomers [19]. A characteristic peak appears at δ = 4.18 ppm, which mainly originates from the proton signals of –CH– and –CH_2_– groups bonded to oxygen atoms in the molecular chains of CMC and PVA, indicating the successful incorporation of oxygen-containing functional groups into the gel system. The simultaneous presence of characteristic signals corresponding to PAM, CMC, and PVA demonstrates that these components coexist within the gel network. Moreover, no obvious resonance peaks associated with vinyl protons of unreacted monomers were detected, indicating that the double bonds of AM were effectively consumed during polymerization. These results confirm that AM underwent successful polymerization and that CMC and PVA were effectively integrated into the crosslinked network, thereby verifying the successful synthesis of the degradable plugging agent.

(3) Rheological Analysis

**Figure 4:**
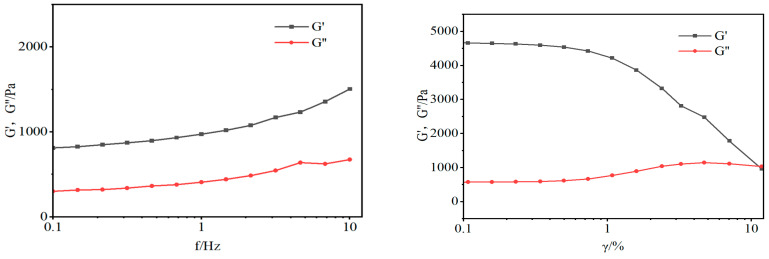
Haake rheometer test results of the degradable gel.

The viscoelastic properties of the degradable gel were evaluated using a HAAKE rheometer by measuring the storage modulus (G′) and loss modulus (G″). The results are presented in Figure 4. Within the strain range of γ = 0.1–1%, the storage modulus G′ remained nearly constant at approximately 4600 Pa and was consistently higher than the loss modulus G″ (approximately 500 Pa), indicating that the gel exhibited predominantly elastic behavior within the linear viscoelastic region. This behavior suggests that the internal network structure remained intact and was capable of effectively resisting external deformation.

When the strain exceeded the critical value of approximately 1%, G′ decreased significantly, while G″ increased and gradually approached G′. This transition indicates the onset of nonlinear deformation and progressive disruption of the gel network structure under increasing strain. Overall, the presence of a stable plateau region with G′ consistently greater than G″ confirms that the material behaves as a typical solid-like gel with a well-developed and stable three-dimensional crosslinked network. The high storage modulus further demonstrates the excellent structural integrity and mechanical strength of the gel, which are beneficial for maintaining effective plugging performance under downhole conditions.

### 3.2. Evaluation of Thermal Resistance

To evaluate the thermal stability of the gel, 3 g of dried gel particles were added to an aging cell containing 400 mL of deionized water. The samples were then aged at 80, 100, and 120 °C, respectively. After aging, the samples were cooled naturally to room temperature, and the gel particles were collected and washed three times with deionized water to remove residual impurities. The swelling ratio was determined in triplicate for each condition, and the results are presented as mean ± standard deviation (Table 2). Subsequently, the strength and toughness of the pre-crosslinked gel particles were evaluated according to the gel strength and toughness classification criteria described in Table 1.

The thermal resistance of gel XZ was evaluated based on its swelling ratio, strength, and toughness after aging in deionized water at different temperatures for 16 h. As shown in Table 2, the swelling ratio of the gel increased with increasing temperature, whereas its strength and toughness exhibited a slight decline. However, the overall changes were relatively small, indicating that the gel network remained largely intact under elevated-temperature conditions.

The increase in swelling ratio can be attributed to enhanced molecular chain mobility and accelerated water diffusion into the gel network at higher temperatures. It should be noted that the pH of the solution was not controlled in this experiment. Since the carboxyl groups (–COOH) of CMC undergo an ionization equilibrium step that is sensitive to pH, variations in pH could affect the electrostatic interactions and swelling behavior of the gel. Future studies should systematically investigate the effect of pH on the swelling kinetics and equilibrium swelling ratio. Although partial weakening of the crosslinked structure occurred, the gel particles maintained good mechanical integrity and deformation resistance after aging. These results demonstrate that the degradable gel possesses excellent thermal stability and can retain favorable swelling and mechanical properties under high-temperature conditions, indicating its suitability for plugging applications in deep coal-measure gas reservoirs.

### 3.3. Evaluation of Salt Resistance

To evaluate the salt resistance of the gel, 400 mL CaCl_2_ solutions with concentrations of 1 wt%, 2 wt%, and 3 wt% were prepared. Subsequently, 3 g of dried gel particles was added to each solution and aged in sealed aging cells at 120 °C for 16 h. After aging, the samples were cooled to room temperature, and the gel particles were recovered and washed three times with deionized water. The swelling behavior, strength, and toughness of the pre-crosslinked gel particles were then evaluated. The swelling ratio was measured in triplicate, and the results are presented as mean ± standard deviation (Table 3). The salt resistance of gel XZ was assessed based on its swelling ratio, strength, and toughness after aging in CaCl_2_ solutions with different salinities at 120 °C for 16 h. As presented in Table 3, the swelling ratio, strength, and toughness of the gel gradually decreased with increasing CaCl_2_ concentration. However, the reductions were relatively small, indicating that the gel maintained its structural integrity and mechanical properties under high-salinity conditions.

The swelling ratio decreased with increasing CaCl_2_ concentration. This decrease can be attributed to the ionic crosslinking effect between Ca^2+^ ions and the carboxylate groups (–COO^−^) on the CMC chains. The divalent Ca^2+^ cations act as ionic crosslinkers, forming additional coordination bonds between adjacent carboxylate groups and restricting chain mobility and network expansion. Simultaneously, the presence of Ca^2+^ reduces the osmotic pressure difference between the gel interior and the external solution, thereby limiting water uptake. Nevertheless, the gel retained favorable swelling capacity and mechanical stability even at elevated salinity, demonstrating excellent salt tolerance and suitability for application in deep coal-measure gas reservoirs with high mineralization levels.

### 3.4. Effect on the Drilling Fluid Rheology

To evaluate the influence of the degradable gel on drilling fluid rheology, 0, 2, 4, 8, and 12 g of gel particles were added to 400 mL of base mud, corresponding to concentrations of 0%, 0.5%, 1%, 2%, and 3%, respectively. The mixtures were stirred at a low speed of 600 rpm for 30 min to ensure uniform dispersion while minimizing particle breakage. The prepared drilling fluids were then transferred into aging cells and hot-rolled at 120 °C for 16 h. After aging, the samples were cooled to below 60 °C and subsequently sheared at 12,000 rpm for 10 min to restore fluid homogeneity. The rheological properties were then measured using a six-speed rotational viscometer (triplicate measurements). The results are presented in Table 4.

As shown in Table 4, both the apparent viscosity (AV) and plastic viscosity (PV) of the drilling fluid increased significantly with increasing gel concentration. Specifically, the AV increased from 2.5 mPa·s to 42 mPa·s, while the PV increased from 2 mPa·s to 40 mPa·s. These results indicate that the degradable gel effectively enhances the viscosity of the drilling fluid system, thereby improving its suspension and cuttings-carrying capacities.

The increase in viscosity can be attributed to the swelling behavior of the gel particles and the formation of a more robust network structure within the drilling fluid. As the gel concentration increased, interactions between the gel particles and the fluid phase became stronger, leading to greater flow resistance and enhanced structural stability. Overall, the degradable gel exhibited a pronounced viscosification effect on the drilling fluid, which is beneficial for improving system stability, wellbore support capability, and plugging performance under downhole conditions.

### 3.5. Evaluation of the Plugging Performance of Gel XZ

**Figure 5:**
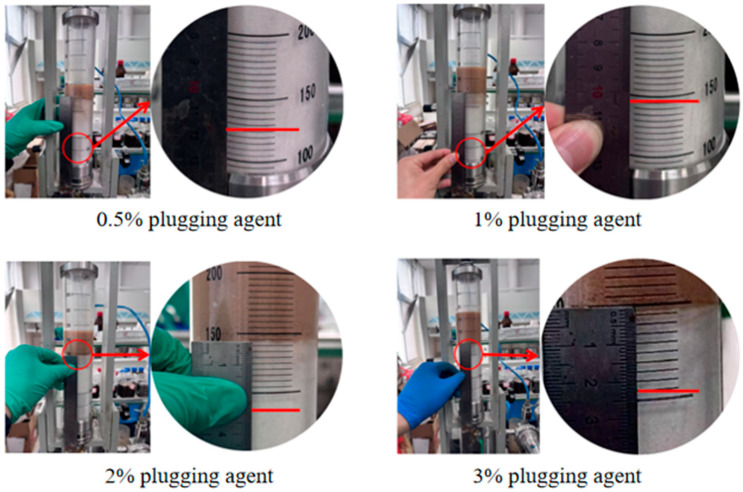
Infiltration depth of sand beds with different concentrations of XZ.

The plugging performance of gel XZ at concentrations of 0.5%, 1%, 2%, and 3% was evaluated using a 2 wt% bentonite-based drilling fluid. The XZ particles were not classified by particle size; instead, the dried gel was directly crushed and used for the plugging tests. It should be noted that the XZ particles were not classified by particle size; instead, the dried gel was directly crushed and used for the plugging tests. Particle size matching with fracture apertures is critical for effective plugging, and the lack of size control in this study represents a limitation. Future work should focus on grinding and sieving to obtain well-defined particle size fractions for optimal sealing performance. The experimental results are presented in Table 5 and Figure 5.

For the 100-mesh sand-bed plugging test, the plugging efficiency improved significantly with increasing XZ concentration. Among the tested concentrations, XZ at 2% and 3% exhibited the best plugging performance, with invasion depths of 2.85 cm and 2.35 cm, respectively. The reduced invasion depth indicates that the swollen gel particles effectively bridged and sealed the pore channels within the sand bed, thereby restricting fluid penetration.

Although the 3% concentration provided the lowest invasion depth, the improvement compared with the 2% concentration was relatively limited. Considering both plugging effectiveness and economic feasibility, a concentration of 2% was selected as the optimal dosage of XZ for subsequent studies. This concentration provides efficient plugging performance while minimizing material consumption, demonstrating its practical potential for wellbore stabilization and formation protection in deep coal-measure gas drilling operations.

### 3.6. Evaluation of the Self-Degradation Behavior of Gel XZ

To evaluate the self-degradation performance of gel XZ, 2 g of dried gel particles and 200 mL of saline solution were transferred into a sealed aging cell and aged at 120 °C for 16 h to simulate downhole formation conditions. After the aging process, the samples were maintained under the specified conditions, and the residual gel mass was measured at two-day intervals. The degradation rate of the gel was calculated using the following equation:

S = (W_1_ − W_2_)/W_1_ × 100% (1)

where W_1_ is the initial mass of the gel before degradation (g), and W_2_ is the residual mass of the gel after degradation for time (t) (g).

As shown in Table 6, gel XZ exhibited excellent self-degradation performance under the simulated formation conditions. The degradation rate increased progressively with degradation time, indicating the gradual breakdown of the gel network structure [20,21]. Notably, the self-degradation rate exceeded 90% by the fifth day, demonstrating that the gel possesses a strong self-removal capability.

The rapid degradation of XZ after fulfilling its plugging function is beneficial for reducing residual plugging material within the formation and minimizing potential damage to reservoir permeability. These results indicate that XZ can effectively balance temporary plugging performance and subsequent degradation, thereby meeting the requirements of formation protection and efficient flowback in deep coal-measure gas drilling operations. Although the degradation products were not identified in this study, the hydrolysis of amide and ether bonds is expected to generate polyacrylamide oligomers and carboxymethyl cellulose fragments. Future work will employ gel permeation chromatography (GPC) and gas chromatography–mass spectrometry (GC–MS) for product identification.

### 3.7. Evaluation of Reservoir Protection Performance

The reservoir protection performance of gel XZ was evaluated using a core-flow apparatus. Artificial cores were first contaminated with drilling fluid containing the degradable gel under simulated drilling conditions. Subsequently, the contaminated cores were immersed in deionized water for 48 h to allow degradation and removal of the plugging material. After immersion, the permeability recovery values of the cores were measured and used to assess the reservoir protection capability of the gel system. The results are presented in Table 7.

As shown in Table 7, the cores treated with the gel-containing drilling fluid exhibited permeability recovery values of approximately 80% after soaking. The relatively high permeability recovery indicates that the degradable gel effectively reduced permanent plugging damage to the pore structure and maintained favorable reservoir flow capacity. This performance can be attributed to the self-degradation behavior of the gel, which enables efficient plugging during drilling operations while allowing subsequent removal after exposure to formation fluids.

### 3.8. Evaluation of Filter-Cake Removal Performance

**Figure 6:**
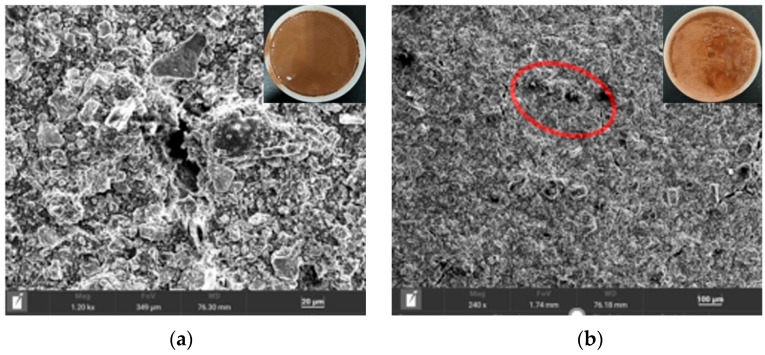
Morphology of the filter cake before and after soaking. (**a**) Before aging. (**b**) After aging.

**Figure 7:**
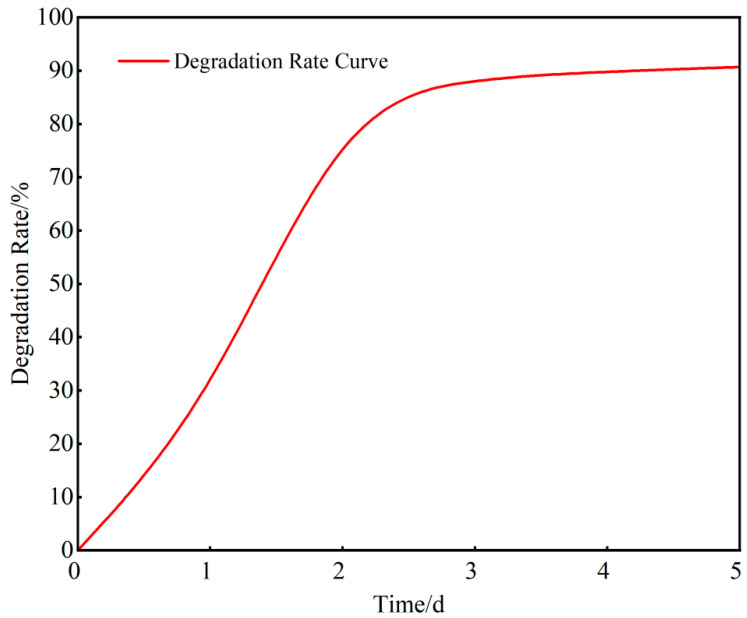
The variation pattern of degradation rate over time of degradation process.

Filter cakes were prepared from the drilling fluid containing the plugging agent using a medium-pressure filtration apparatus. The filter cakes were then immersed in deionized water at 100 °C for 48 h, and the changes in the filter cakes were observed (Figure 6). After soaking in water, the XZ contained in the filter cake underwent self-degradation, resulting in a significant reduction in filter-cake thickness and demonstrating good self-removal capability. XZ exhibited excellent water-swelling performance, plugging capacity, strength, and toughness. Although particle size can be adjusted in principle according to the dimensions of loss channels, the current study did not implement size classification, and this aspect requires further optimization. In addition, XZ showed good temperature and salinity tolerance. The degradation rate increased with temperature, while inorganic salts had little effect on the degradation process. The degradation rate exceeded 90% on the fifth day, indicating rapid removal after plugging (Figure 7). The filter cake formed by the XZ-containing drilling fluid became noticeably thinner after water immersion, and the permeability recovery of the treated core reached approximately 80%, demonstrating that XZ possesses efficient plugging, self-removal, and reservoir protection capabilities and can effectively reduce formation damage.

### 3.9. Evaluation of Drilling Fluid Performance

As shown in Table 8, the rheological properties of the drilling fluid were significantly improved after the addition of the plugging agent. The apparent viscosity (AV), plastic viscosity (PV), and yield point (YP) increased from 19.5, 15, and 4.5 to 62, 42, and 16, respectively, indicating enhanced cuttings-carrying and suspension capacities of the drilling fluid system.

Meanwhile, the API fluid loss decreased from 6.0 mL to 4.4 mL, demonstrating the good fluid-loss control performance of the plugging agent. After aging at 120 °C for 16 h, the API fluid loss further decreased to 3.6 mL, indicating that the plugging agent possesses good thermal stability and sustained plugging capability. These results suggest that the plugging agent can effectively improve the rheological properties of the drilling fluid and reduce fluid loss.

**Figure 8:**
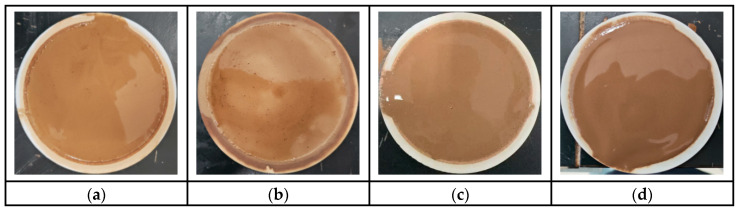
Filter-Cake Morphology Analysis. (**a**) Before aging. (**b**) After aging. (**c**) Adding the sealing agent before aging. (**d**) After aging with the addition of the sealing agent.

Figure 8 shows the changes in the morphology of drilling-fluid filter cakes before and after the addition of the plugging agent. The filter cakes formed by the base drilling fluid exhibited relatively rough and locally non-uniform surfaces both before and after aging. In contrast, after the addition of the plugging agent, the filter cakes became smoother, denser, and more continuous. After aging at 120 °C for 16 h, the drilling fluid containing the plugging agent still formed an intact and compact filter cake, indicating that the plugging agent can effectively improve filter-cake quality and enhance both plugging performance and high-temperature stability.

## 4. Conclusions

(1) FTIR results confirmed that AM, CMC, and PVA were successfully incorporated into the gel system, forming a stable multiple-crosslinked network structure. Thermogravimetric analysis showed that the degradable gel XZ possessed good thermal stability, with an initial decomposition temperature (T_0_) of 116 °C and a half-decomposition temperature (T_1_/_2_) of 445 °C. The covalently crosslinked network formed by AM and the crosslinking agent, together with the ionic crosslinked structure of CMC, provided the gel with a robust three-dimensional framework and excellent water-swelling capacity.

(2) XZ exhibited strong water-swelling ability, excellent plugging capacity, and superior mechanical strength and toughness. Although particle size can be adjusted in principle to match loss-channel dimensions, the current study did not implement size classification—a limitation that should be addressed in future work through controlled grinding and sieving. Thermal- and salt-resistance tests demonstrated that XZ possessed good temperature and salinity tolerance. The degradation rate increased with temperature, while inorganic salts had little influence on the self-degradation process, indicating good adaptability to formation-water environments.

(3) The addition of XZ significantly increased the viscosity of the drilling fluid and reduced fluid loss, demonstrating excellent plugging performance. Meanwhile, XZ showed good self-degradation characteristics, with a degradation rate exceeding 90% on the fifth day. After degradation, the filter cake became noticeably thinner, and the permeability recovery of the core reached approximately 80%, indicating effective reservoir protection.

(4) Drilling-fluid performance evaluations before and after aging demonstrated that XZ could effectively improve the overall performance of the drilling fluid. While maintaining system stability, it significantly reduced fluid loss and enhanced plugging capacity, with fluid-loss control showing the most pronounced improvement.

## Figures and Tables

**Table 1:** Classification Criteria for Gel Strength and Toughness.

Grade	Strength	Toughness
A	Semi-fluid gel with extremely low strength	Extremely poor toughness; highly susceptible to fracture and fragmentation
B	Soft gel with slight elasticity	Poor toughness; easily fractured and fragmented
C	Deformable gel with low elasticity	Moderate toughness; moderate resistance to fracture and fragmentation
D	Deformable gel with moderate elasticity	Good toughness; not easily fragmented
E	Deformable gel with high elasticity	Excellent toughness; difficult to fracture and fragment

**Table 2:** Results of the Gel Thermal Stability Test (mean ± SD, n = 3).

Temperature/°C	Swelling Ratio	Strength Grade	Toughness
80	35.58 ± 1.2	C	C
100	59.51 ± 1.8	C	B
120	70.34 ± 2.1	B	B

**Table 3:** Results of the Gel Salt Resistance Performance Test (mean ± SD, n = 3).

Concentration/%	Swelling Ratio	Strength Grade	Toughness
1	11.8 ± 0.9	D	C
2	10.6 ± 0.7	D	B
3	9.9 ± 0.6	C	B

**Table 4:** Rheological Properties of the Base Slurry after the Addition of the Plugging Agent (mean ± SD, n = 3).

Concentration/%	AV	PV	YP	10-min/10-s Gel Strength Ratio
0	2.5 ± 0.2	2 ± 0.1	0.5 ± 0.1	0.5/0.25
0.5	4.5 ± 0.3	4 ± 0.2	0.5 ± 0.1	0.5/0.5
1.0	14 ± 0.5	13.5 ± 0.5	0.5 ± 0.1	2/2
2.0	28 ± 0.8	21.5 ± 0.7	7 ± 0.3	2.5/3
3.0	42 ± 1.0	40 ± 1.0	2 ± 0.2	1/1

**Table 5:** Experimental Results of Sand Bed Sealing with XZ 100-mesh Sand Layers at Different Concentrations (invasion depth, cm; mean ± SD, n = 3).

Concentration (%)	0.5	1	2	3
Infiltration depth (cm)	11.7 ± 0.4	10.4 ± 0.3	2.85 ± 0.1	2.35 ± 0.08

**Table 6:** Self-degradation rate of XZ under simulated stratum conditions (mean ± SD, n = 3).

Time (d)	1	2	3	4	5
Self-degradation rate (%)	27.16 ± 1.5	83.86 ± 2.0	88.60 ± 1.8	89.84 ± 1.5	90.69 ± 1.2

**Table 7:** Permeability Recovery Experiment (mean ± SD).

Core	Initial Permeability (mD)	Permeability After Soaking (mD)	Recovery (%)
1	110.5	89.7	81.2 ± 1.5
2	105.1	83.9	79.8 ± 1.6
3	111.8	90.1	80.6 ± 1.4

**Table 8:** The Effects of Adding a Sealant on the Rheological and Filtration Properties of the Drilling Fluid System (mean ± SD, n = 3).

Formulation	120 °C × 16 h	AV/(mPa·s)	PV/(mPa·s)	YP/Pa	API/mL	Thickness/mm
Original formulation	Before	19.5 ± 0.6	15.0 ± 0.5	4.5 ± 0.3	6.0 ± 0.2	0.98 ± 0.02
	After	9.0 ± 0.4	7.0 ± 0.3	2.0 ± 0.2	4.4 ± 0.2	0.50 ± 0.03
Original formulation + plugging agent	Before	62.0 ± 1.2	42.0 ± 1.0	16.0 ± 0.8	4.4 ± 0.2	1.20 ± 0.05
	After	—	—	—	3.6 ± 0.2	1.90 ± 0.06

## Data Availability

The original contributions presented in this study are included in the article. Further inquiries can be directed to the corresponding author.
